# Serologic Evidence of Human and Swine Influenza in Mayan Persons

**DOI:** 10.3201/eid1101.040554

**Published:** 2005-01

**Authors:** Guadalupe Ayora-Talavera, Juan Manuel Cadavieco-Burgos, Alejandro Bernardino Canul-Armas

**Affiliations:** * Universidad Autónoma de Yucatán, Mérida, Yucatan; Serologic Evidence of Human and Swine Influenza

**Keywords:** Influenza, serology, HI test, human antibodies, Yucatan, Mayan, dispatch

## Abstract

Antibodies against influenza viruses were detected in 115 serum samples from indigenous Mayan persons from Kochol, Yucatán. Seropositivity rates were 26.9% to A/Bayern/7/95, 40.8% to A/Sydney/5/97, 1.7% to A/Swine/Wisconsin/238/97, and 79.1% to A/Swine/Minnesota/593/99. This report is the first in Mexico of the prevalence of antibodies to swine influenza virus in humans.

Influenza virus type A has the capacity to infect humans, birds, swine, and other animals. Studies have repeatedly shown that influenza virus can move from 1 species to another. The pig has been proposed as an animal that could play a key intermediary role in interspecies transmission. Pigs are the only domesticated mammalian species that are reared in abundance and are susceptible to both avian and human influenza virus and allow productive viral replication (*1*,*2*).

In rural zones in the Mexican state of Yucatán, the “backyard system,” a production system in which animals such as pigs, ducks, turkeys, and chickens are all raised in close proximity to humans, is common. This system is a traditional activity of indigenous Mayan persons, as well as other ethnic groups in Mexico, and provides an economical way to produce animals. The animals eat, live, and share space, water sources, and even food with humans; they may even be found inside houses. These activities create health concerns because of potential for the adaptation and reassortment of human and avian viruses.

Despite abundant evidence supporting interspecific transmission and genetic reassortment of influenza virus around the world, little is known about the influenza virus in humans and domesticated animals in Yucatán in southeastern Mexico. We describe serologic evidence of antibodies against influenza strains from humans and pigs in indigenous Mayan persons from Yucatán.

## The Study

Kochol is located in east Yucatán, ≈20 km from the municipality of Maxcanu. The 1,207 residents are mostly dedicated to agricultural activities (*3*). The population has high illiteracy rates, poor environmental health, and crowded and inadequate housing. In Kochol, pigs are found around the town, walking in and out of houses. All pigs are wild or *criollos*. Some families have 1–18 pigs. For this study, serum samples from 115 persons were made available by the health official of Kochol in 2000. Serum samples were from Kochol residents who came to the health service for any medical condition and required laboratory tests.

Samples were treated with receptor-destroying enzyme from *Vibrio cholera* and heated at 56°C in a water bath to inactivate nonspecific inhibitors (*4*). The following 4 influenza strains were used to detect antibodies: A/Swine/Wisconsin/238/97 (classical swine H1N1), A/Bayern/7/95 (human H1N1), A/Sydney/5/97 (human H3N2), and A/Swine/Minnesota/593/99 (reassortant swine H3N2); all were grown in 10-day-old embryonated chicken eggs. The hemagglutination inhibition tests were performed by using chicken erythrocytes at a concentration of 0.5%. A sample was considered seropositive to H1 and H3 when the HA titer was >1:40. Each serum sample was tested against chicken receptor–destroying enzymes in the absence of virus to rule out induction of nonspecific hemagglutination.

## Conclusions

As shown in Table 1, reactivity rates were uniformly high to H3 subtype influenza virus. These results agree with previous serologic tests of human serum samples from Yucatán (G. Ayora-Talavera, unpub. data). H1 viruses likely circulate at a lower frequency than H3 viruses. Overall, 31 (26.9%) of 115 samples were positive to H1, whereas 93 (80.8%) of 115 were seropositive to H3. The results indicate that influenza virus infection occurs in a large proportion of persons in this area. In general, Mexican persons are not vaccinated, so we can be sure that the antibodies detected reflect actual infection (*5*). Samples were divided into 5 age groups (Table 2). By analyzing the percentage of seropositive persons in different age groups, we observed that persons 15–24 years of age were most commonly seropositive. Through virus surveillance in Yucatán, we have also observed a very low circulation of influenza A H1. From ≈1,500 throat swabs collected in 5 years, no sample has been found to contain H1 influenza by immunofluorescence assay, and only 5 viruses have been detected with reverse transcription–polymerase chain reaction (G. Ayora-Talavera, unpub. data).

**Table 1:** Hemagglutination inhibition antibodies to influenza virus, Kochol, Yucatán

No. samples	Titer	Month	No. (%) positive samples			
			A/Bayern/7/97 (H1N1)	A/Sw/Wis/238/97 (H1N1)	A/Sw/Mn/593/99 (H3N2)	A/Sydney/5/97 (H3N2)
73		June	22 (30)	2 (2.7)	59 (80.8)	32 (43.8)
	0		41	67	4	10
	10		6	2	2	12
	20		4	2	8	19
	40		5	2	9	9
	80		6		11	17
	160		7		18	4
	320					2
	640		4		18	
	1,280				3	
35		July	8 (23)	0	26 (74)	14 (40)
	0		18		5	13
	10		6		2	2
	20		3		2	6
	40		3		7	8
	80		3		10	4
	160		1		4	2
	640		1		4	
	1,280				1	
7		August	1 (14)	0	6 (85.7)	1 (14)
	0		4		1	3
	10		1			1
	20		1			3
	40		1		1	1
	160				4	
	320				1	

**Table 2:** Specific hemagglutination inhibition antibodies by age group, Kochol, Yucatán

Age group/total	N	n (%)			
8–14	16	A/Bayern/7/97 (H1N1)	A/Sw/Wis/238/97 (H1N1)	A/Sw/Mn/593/99 (H3N2)	A/Sydney/5/97 (H3N2)
15–24	33	4 (25)	0	14 (87)	9 (56)
25–34	28	13 (39)	0	29 (88)	14 (42)
35–44	24	5 (16)	0	22 (78)	9 (32)
45–53	14	4 (16)	1 (4)	16 (66)	9 (37)
Total		4 (33)	1 (8)	10 (71)	6 (43)

The highest seropositivity rates across all age groups were detected with the A/Sw/Minnesota virus as antigen. Although this strain was isolated from American pigs, the HA, NA, and PB1 genes are of human origin (*6*). Taking into consideration the cutoff values of this study, seropositivity to the swine H1 virus was only detected in 2 samples, from persons 43 and 59 years of age. However, lower titers were detected in 4 more persons 33–55 years of age. The weak reactivity to this virus could suggest a past exposure of adult persons to viruses of swine origin, a situation that has not occurred in persons >30 years of age.

The animal population owned by persons in this study consisted of pigs (68.7%), chickens (73%), and ducks (17.3%). Any combination of 2 or 3 species was kept by 54.7%. The range of the number of animals owned was 0–12 (mean 2.9) pigs, 0–60 (mean 7) chickens, and 0–23 (mean 0.93) ducks. Since we did not have avian antigens available, serum samples collected from humans, pigs, chickens, and ducks were not tested for exposure to avian influenza viruses.

The relative risk of being seropositive for H1 or H3 viruses from exposure to pigs was 1.93 with human H1 (95% confidence interval [CI] 1.2–3.0), 0.88 with human H3 (95% CI 0.55–1.4), 0.6 with swine H1 (95% CI 0.08–4.2), and 1.0 with swine H3 (95% CI 0.62–1.6).

Serologic evidence of swine antibodies in persons in contact with pigs has been reported in several studies (*7*–*12*). In Mexico, apart from this report, no information about the prevalence of antibodies to swine influenza virus in humans exists. The only information available comes from a study carried out on pig farms in central Mexico, where the subtype H1 is prevalent in 20% of pigs (*13*) and from a previous study from Yucatán, where the most prevalent subtype in pig farms is H3 (65%) and H1 (20%) (*14*).

As a result of the Mexican outbreak of HPAI H5N2, the Mexican Ministry of Agriculture (SAGARPA) implemented a national surveillance system in all chicken farms (NOM-044-ZOO-1995). Yucatán is considered a free state for avian influenza virus. Chicken farms are sampled 3 times a year for serologic surveillance, and 10% of the backyard flocks are sampled annually (*15*). On the other hand, swine influenza is not considered within the SAGARPA priorities, and no surveillance program exists for swine farms, although we found serologic evidence that in Yucatán influenza H3 subtype is highly prevalent (*14*).

Asia has been considered as an epicenter for the generation of pandemic influenza virus, and some factors are high densities of humans and animals in close contact (*1*). In Yucatán, the backyard system is a common practice, and human and animal encounters could lead to generation of novel reassortant viruses here as well.
